# Bimetallic Deep Eutectic Solvent-Driven Ce-Fe Oxide Nanozyme Based on Electron Transfer for the Colorimetric Detection of *E. coli* O157:H7 in Food

**DOI:** 10.3390/foods15081391

**Published:** 2026-04-16

**Authors:** Luyang Zhao, Yang Song, Guoyang Xie, Hengyi Xu

**Affiliations:** State Key Laboratory of Food Science and Resources, Nanchang University, Nanchang 330047, China; 357900230020@email.ncu.edu.cn (L.Z.); wangsyang202011@163.com (Y.S.); 212301006@csu.edu.cn (G.X.)

**Keywords:** *E. coli* O157:H7, deep eutectic solvents, bimetal oxide nanozyme, electron transfer, colorimetric biosensor

## Abstract

Sensitive detection of *Escherichia coli* O157:H7 (*E. coli* O157:H7) in food matrices remains an important analytical challenge. Here, a colorimetric biosensor was constructed based on a bimetal oxide nanozyme composed of Ce-Fe oxide. This biosensor achieved sensitive detection of *E. coli* O157:H7. The Ce-Fe oxide synthesized on the basis of deep eutectic solvents (DESs) had the advantages of low solvent consumption and short preparation time. By regulating the two key factors of metal valence and oxygen vacancy content, the peroxidase (POD) activity of the nanozyme was significantly improved. Compared with the single-metal oxide nanozyme Fe oxide, the addition of Ce increased the Fe^2+^/Fe^3+^ ratio from 0.37 to 0.49, implying a possible enhancement of electron transfer between Fe^2+^ and Fe^3+^. The detection limits (LODs) of the biosensor based on Fe oxide and that based on Ce-Fe oxide were 10^2^ CFU/mL and 10^1^ CFU/mL, respectively, comparable to existing validated methods. Moreover, these two biosensors achieved satisfactory recovery rates (91–104%) and RSDs (1.2–8.8%) in the spiked lake water, juice, and lettuce samples of *E. coli* O157:H7, indicating their high potential for application in spiked sample detection. In summary, the method proposed in this study for improving the POD activity of nanozymes through electron transfer in DES solutions is beneficial to the development of metal oxide nanozymes.

## 1. Introduction

Ensuring food safety has become a paramount global concern, with foodborne pathogens presenting an emergent threat that necessitates advanced detection and control strategies. Various countries have proposed targeted laws and regulations on how to detect foodborne pathogens. Foodborne pathogens can cause food poisoning or spread through food as a medium [1,2]. According to the World Health Organization (WHO) estimates, approximately 600 million people develop foodborne illness annually, resulting in about 420,000 deaths [3]. *Escherichia coli* O157:H7 (*E. coli* O157:H7), as one type of foodborne pathogen, presents initial symptoms of non-bloody diarrhea after infection. Some patients may develop bloody diarrhea in the later stage [4]. In the United States, *E. coli* O157:H7 is one of the most prevalent etiological agents of hemolytic uremic syndrome [5]. Therefore, it is imperative to develop a simple, portable, specific and sensitive detection technology to detect *E. coli* O157:H7 [6].

The dominant cultivation methodologies, encompassing pre-enrichment, isolation, and biochemical identification, are recognized as the standard protocol for detecting *E. coli* O157:H7 due to their high accuracy. However, this cultivation method is time-consuming and cumbersome, requiring 48–72 h per batch, while only identifying a limited range of microorganisms and necessitating professional operators [7]. To overcome these drawbacks, various rapid detection techniques have emerged, including molecular methods such as polymerase chain reaction (PCR) and loop-mediated isothermal amplification (LAMP), immunological assays, and aptamer (Apt)-based colorimetric sensors [8]. Nevertheless, each approach still faces specific limitations: molecular methods demand costly instrumentation and skilled personnel, antibodies suffer from poor storage stability and high cost [9], and Apt-based sensors alone often lack sufficient catalytic signal amplification for sensitive detection [10]. Consequently, there remains a need to establish a rapid, convenient, and widely applicable detection method.

Aptamers serve as excellent recognition elements for colorimetric detection due to their high specificity, chemical stability, and ease of synthesis [10,11]. However, Apt-target binding alone does not produce a strong colorimetric signal; an efficient signal amplification strategy is required. Nanozymes, which are nanomaterial-based enzyme mimics, offer a promising solution [12]. In comparison with natural enzymes, nanozymes offer certain advantages, including cost-effectiveness, enhanced stability, tunable surface properties, ease of synthesis, and ease of storage [13,14]. Among the diverse family of nanozymes, metal oxide nanostructures have garnered significant research interest due to their highly tunable properties. By precisely modulating their composition, morphology, size, and surface valence states, the catalytic performance and selectivity of metal oxide nanozymes can be tailored to meet specific application requirements [15]. For instance, Fe3O4 nanoparticles are widely studied for their excellent biocompatibility and intrinsic magnetic properties, which facilitate targeted delivery and separation in biomedical applications [16]. Meanwhile, ZnO nanostructures exhibit a suite of advantageous characteristics, including high optical transparency, remarkable electron mobility, and robust chemical stability, making them attractive for sensing and catalytic uses [17]. Furthermore, the ease of synthesis, low toxicity, and natural abundance of ZnO have established it as an important carrier material in nanozyme design [18]. Despite these advantages, the rational design and synthesis of metal oxide nanozymes with well-defined properties remain challenging due to the complexity and limited controllability of conventional synthesis routes. These routes often rely on toxic organic solvents, high temperatures, and hazardous reagents, raising environmental and safety concerns. Therefore, exploring green and sustainable synthesis strategies is of great importance.

Deep eutectic solvents (DESs) are eutectic mixtures of hydrogen bond acceptors and donors, characterized by tunable physicochemical properties and low environmental impact. In this study, DESs were exploited beyond their role as green solvents; they actively influenced the synthesis of Ce-Fe oxide nanozymes through three specific mechanisms. First, the hydrogen-bond network of DESs provides a well-defined coordination environment. Carboxyl-rich donors such as citric acid chelate Ce^3+^ and Fe^3+^ ions, ensuring homogeneous metal distribution and promoting Ce-Fe interaction. Second, the viscosity of DESs modulates nucleation kinetics. The viscous medium slows diffusion, enabling controlled growth and facilitating the formation of oxygen vacancies and surface defects—key determinants of peroxidase (POD) activity. Third, the DES-based synthesis operates under mild, energy-efficient conditions (80 °C, no organic solvents), meeting green chemistry criteria. While DESs have been used to prepare various functional materials [19,20,21,22,23], their application in synthesizing peroxidase mimics is rarely reported. Our work thus demonstrates a DES-mediated strategy to engineer bimetallic oxide nanozymes with enhanced catalytic performance.

Against this background, the present study aimed to develop a bimetallic oxide nanozyme (Ce-Fe oxide) using DESs as a green and tunable synthesis platform. We hypothesized that the incorporation of Ce into the Fe oxide matrix could modulate the metal valence states and oxygen vacancy content, thereby enhancing the POD activity of the nanozyme. Furthermore, we sought to integrate this nanozyme into a colorimetric biosensor for the specific detection of *E. coli* O157:H7. Compared with a single-metal Fe oxide-based sensor, the Ce-Fe oxide-based platform is expected to offer improved analytical performance, including higher sensitivity and better reproducibility. The DES-assisted synthesis also offers advantages such as reduced solvent consumption and milder reaction conditions, which may facilitate scalable fabrication of nanozyme-based biosensors.

## 2. Materials and Methods

### 2.1. Materials and Reagents

The aptamer sequences used for *E. coli* O157:H7 recognition was 5’-NH_2_-CCG GAC GCT TAT GCC TTG CCA TCT ACA GAG CAG GTG TGA CGG-3’, synthesized by Shanghai Sangon Biotech Co., Ltd. (Shanghai, China). Carboxylated magnetic nanoparticles (180 nm) was purchased from Allrun Nano (Shanghai, China). Luria-Bertani medium was bought from Beijing Land Bridge (Beijing, China). Agar powder, benzoic acid (BA) and methylene blue (MB) were purchased from Solarbio (Beijing, China). 3,3’,5,5’-Tetramethylbenzidine (TMB) was purchased from Sangon Biotech (Shanghai, China). 30% hydrogen peroxide (H_2_O_2_), 1-Ethyl-3-(3-dimethyllaminopropyl) carbodiimide hydrochloride (EDC), nhydroxysulfosuccinimide sodium salt (NHSS), Cerium (III) nitrate hexahydrate Ce(NO_3_)_3_·6H_2_O and Dimethyl sulfoxide (DMSO) were purchased from Aladdin (Shanghai, China). Iron chloride hexahydrate and 20×phosphate buffered saline (20×PBS) buffer solution were all provided by Macklin (Shanghai, China). Sodium hydroxide (NaOH) and citric acid was from Sinopharm (Shanghai, China). Anhydrous sodium acetate and acetic acid were purchased from Xilong Scientific (Shantou, China). Ultrapure water used in all experiments was from the Milli-Q system (Millipore, Burlington, MA, USA). All glassware was soaked in aqua regia (HNO_3_:HCl = 1:3, *v/v*) for more than 2 h and then rinsed thoroughly with ultrapure water. All chemicals were at least analytical reagent grade.

### 2.2. Apparatus

The morphology of Ce-Fe oxide was investigated using a Scanning Electron Microscope (SEM) (Regulus 8100, Hitachi, Japan). Powder X-ray Diffraction (XRD) measurements were performed using a diffractometer (Bruker D8 Advance, Karlsruhe, Germany). Surface chemical elements were tested by X-ray photoelectron spectroscopy (Thermo Scientific K-Alpha, Waltham, MA, USA). The chemical functional groups were characterized by the Fourier transform infrared spectroscopy (FT-IR) (Thermo Fisher Scientific Nicolet iS20, Waltham, MA, USA). The UV-vis absorbance spectra were obtained by a microplate S-4 reader (Varioskan multimode microplate reader, Thermo Fisher Scientific, Waltham, MA, USA).

### 2.3. DESs-Assisted Synthesis of Ce-Fe Oxide

The synthesis of Ce-Fe oxide was slightly modified based on the reference method [24]. To prepare the DESs, 43.4 mg of Ce(NO_3_)_3_·6H_2_O (100 μmol) and 19.2 mg of citric acid (100 μmol) were mixed and heated at 80 °C for 30 min until a liquid DES formed. Then, 27 mg of FeCl_3_·6H_2_O (100 μmol) was added to the DESs and stirred at 600 rpm at 80 °C for 1 h to ensure complete dissolution. Subsequently, 140 μL of 5 M NaOH solution was added dropwise under vigorous stirring (600 rpm), and the mixture was further stirred at 80 °C for 3 h to facilitate the formation of Ce-Fe oxide. After the reaction, the product was collected by centrifugation at 8000 rpm for 5 min, then washed three times with deionized water (10 mL per wash, each wash followed by centrifugation at 8000 rpm for 5 min). The stock was diluted 150-fold to a working concentration of ~0.8 g/mL. Only a 1:1 Ce: Fe molar ratio was used. To evaluate the activity of individual metal components, Mn oxide, Co oxide, Ce oxide, and Fe oxide were synthesized under identical conditions. To investigate the influence of different metal dopants on the activity of Ce-Fe oxide, Fe-Mn oxide and Fe-Co oxide were prepared. Furthermore, to examine the effect of the addition sequence of bimetal compounds on POD activity, Fe-Ce oxide, Mn-Fe oxide, and Co-Fe oxide were synthesized.

### 2.4. POD Activity Analysis of Fe Oxide and Ce-Fe Oxide

Subsequently, enzymatic kinetics analysis was conducted on Fe oxide and Ce-Fe oxide to evaluate their POD activity. All experiments were performed in triplicate to ensure reproducibility, and the data were expressed as mean ± standard deviation to reflect the variability of kinetic parameters. The specific steps are as follows: 190 μL of sodium acetate buffer solution (NaAc, pH = 3.6), different concentrations of 3,3′,5,5′-tetramethylbenzidine (TMB) solution (0.1, 0.15, 0.2, 0.25, 0.3, 0.35, 0.4 mM), and 10 μL of Fe oxide or Ce-Fe oxide solution (0.8 mg/mL) were mixed with 25 μL of different concentrations of H_2_O_2_ solution (0.5, 1, 5, 10, 15, 20, 25 mM). The mixture was vortexed thoroughly and incubated at room temperature for 5 min to allow the catalytic reaction to reach a steady state. Following the 5 min reaction, the absorbance value at 652 nm was immediately measured using a UV-Vis spectrophotometer, and the initial reaction velocity was calculated based on the linear increase in absorbance within the first 1 min to avoid the influence of substrate depletion and product inhibition. The kinetic parameters were determined by fitting the experimental data to the Lineweaver–Burk double reciprocal equation, which is the linear transformation of the Michaelis–Menten equation:

(1) $$\frac{1}{v}\;=\;\frac{K_{m}}{V_{\max}}\;\times \;\frac{1}{\left[S\right]}\;+\;\frac{1}{V_{\max}}$$

where V_max_ represents the maximum initial velocity of the catalytic reaction; [S] denotes the concentration of the substrate (TMB or H_2_O_2_) in the steady-state kinetic analysis; and K_m_ is the Michaelis constant, which reflects the affinity of the nanozyme for the substrate.

The specific activity (SA) of each nanozyme was calculated using the following formula:

(2) SA (μmol·mg^−1^·min^−1^) = (ΔA × V_total_ × 10^6^)/(ε × m_enzyme_ × t)

where ΔA is the increase in absorbance at 652 nm within the initial linear period (first 2 min), V_total_ is the reaction volume (0.25 mL), ε is the molar extinction coefficient of oxidized TMB (39,000 M^−1^·cm^−1^ at 652 nm), m_enzyme_ is the mass of nanozyme in the reaction mixture (mg), and t is the reaction time (1 min). All SA values are expressed as mean ± SD from three independent experiments.

### 2.5. Colorimetric Detection of E. coli O157:H7

For Apt immobilization, two conjugates were prepared via covalent coupling using EDC/NHSS chemistry. To prepare MBs@Apt, 100 μL of carboxylated magnetic beads (10 mg/mL) were activated with 29 μL of 10 mg/mL EDC and 32.5 μL of 10 mg/mL NHSS in 940 μL PBS (pH = 7.4) at 37 °C for 1 h. After washing twice, 50 μL of 8 μM 5′-NH_2_-Apt (pre-denatured at 95 °C for 5 min) was added and incubated at 37 °C for 6 h, followed by three washes with PBS; the final product was resuspended in 1 mL PBS. To prepare Ce-Fe oxide@Apt, 100 μL of Ce-Fe oxide (0.8 mg/mL) was activated with 100 μL each of 10 mg/mL EDC and NHSS at 37 °C for 1 h, then 10 μL of 10 μM Apt was added and incubated at 37 °C for 3 h, after which the mixture was centrifuged (8000 rpm, 5 min) and washed twice with PBS, then resuspended in 1 mL PBS.

The sandwich assay was performed as follows. In a 1.5 mL tube, 850 μL PBS, 50 μL MBs@Apt, and 100 μL of *E. coli* O157:H7 at various concentrations were incubated at 37 °C for 40 min. The tube was then placed on a magnetic rack, the supernatant removed, and the pellet washed three times with 1 mL PBS each (resuspending by vortexing and re-separating). To block non-specific binding sites, 100 μL of 10% bovine serum albumin (BSA) and 900 μL PBS were added, incubated at 37 °C for 30 min, followed by two washes with PBS. The captured bacteria were resuspended in 200 μL PBS, and 50 μL of Ce-Fe oxide@Apt was added; the mixture was incubated at 37 °C for 30 min to form the sandwich complex (MBs@Apt-*E. coli* O157:H7-Apt@Ce-Fe oxide). After three additional washes with PBS, the final complex was resuspended in 100 μL PBS. For colorimetric detection, 100 μL NaAc buffer (0.1 M, pH = 3.6), 25 μL TMB (2.5 mM), and 25 μL H_2_O_2_ (10 M) were added. After incubation at 25 °C for 5 min, the absorbance at 652 nm was measured. A standard curve was constructed by plotting A_652_ against the logarithm of bacterial concentration (CFU/mL). Three biological replicates (independently prepared sensors and fresh bacterial cultures) were performed, each with three technical replicates. The limit of detection (LOD) was calculated as 3σ/S, where σ is the standard deviation of ten blank samples, and S is the slope of the calibration curve.

### 2.6. Spike and Recovery Test in Food Matrices

To preliminarily evaluate the feasibility of the developed biosensor in complex matrices, spiked recovery tests were conducted using three types of sample matrices: lettuce, orange juice, and lake water. These samples were purchased from a local market (lettuce and orange juice) or collected from Nanchang University (lake water). Lettuce samples were ground in a mortar, dissolved in PBS, and filtered to remove insoluble impurities. Orange juice and lake water were directly filtered. All three samples were then sterilized at 121 °C under high pressure for 15 min to eliminate background microorganisms that could interfere with the detection. After sterilization, 4 mL of each sample was transferred into sterile culture tubes. *E. coli* O157:H7 suspensions at various concentrations were spiked into the tubes, and the mixtures were incubated at 37 °C for 4 h [25]. This incubation step was necessary to allow the bacteria to recover and proliferate to detectable levels, given the low initial spiking concentrations (10^2^–10^4^ CFU/mL). Following incubation, the absorbance at 652 nm was measured according to the procedure described in Section 2.5, and recovery rates as well as RSD values were calculated. Recovery (%) = (measured concentration/spiked concentration) × 100%. Three biological replicates (each with three technical replicates) were performed. It should be noted that these experiments were performed in sterilized, spiked matrices rather than naturally contaminated samples, and the 4 h enrichment step limits the ability to claim real-time or field applicability. Therefore, the results should be interpreted as a proof-of-concept validation of the biosensor’s potential utility under simplified conditions.

### 2.7. Statistical Analysis

All experiments were performed with at least three independent biological replicates, and data are presented as mean ± standard deviation (SD). Statistical analysis was performed using one-way analysis of variance (ANOVA) followed by Tukey’s HSD post hoc test for multiple comparisons. A compact letter display (CLD) was used to denote statistically significant differences among groups: groups sharing the same letter are not significantly different (*p* > 0.05), while groups with different letters indicate significant differences (*p* < 0.05). All analyses were conducted using OriginPro 2024b (OriginLab, Northampton, MA, USA).

## 3. Results

### 3.1. Principle of the Colorimetric Biosensor Based on Ce-Fe Oxide for E. coli O157:H7 Detection

**Figure 1:**
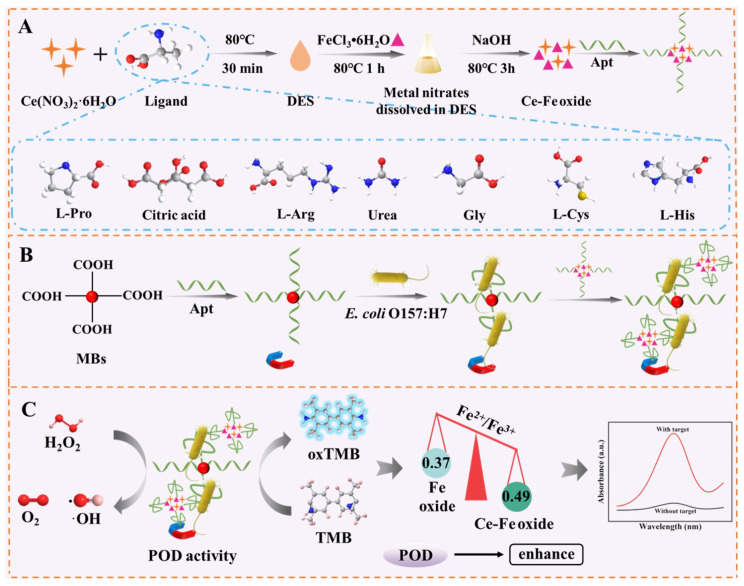
Schematic illustration of the sensing platform based on Ce-Fe oxide for *E. coli* O157:H7 detection. (**A**) Preparation of Ce-Fe oxide. (**B**) The formation of MBs and sandwich structures. (**C**) Colorimetric detection of *E. coli* O157:H7 and mechanism of action.

A schematic illustration of the working principle is presented in Figure 1. Briefly, the Ce-Fe oxide nanozyme was synthesized via a DES-assisted method, which generates abundant oxygen vacancies and Fe^2+^, conferring strong POD activity. The Apt serves as the recognition element. Upon addition of *E. coli* O157:H7, a sandwich assembly—MBs@Apt/*E. coli* O157:H7/Apt@Ce-Fe oxide—was formed, enabling magnetic capture and colorimetric signal generation. The successful combination of Apt and Ce-Fe oxide was demonstrated through Zeta potential (Figure S1). In the presence of H_2_O_2_ and TMB, the nanozyme catalyzes the production of hydroxyl radicals (·OH) that oxidize TMB to a blue product (oxTMB) with an absorbance peak at 652 nm. The absorbance decreases proportionally with increasing bacterial concentration, allowing quantitative analysis in food matrices.

### 3.2. Preparation and Screening of DESs

**Figure 2:**
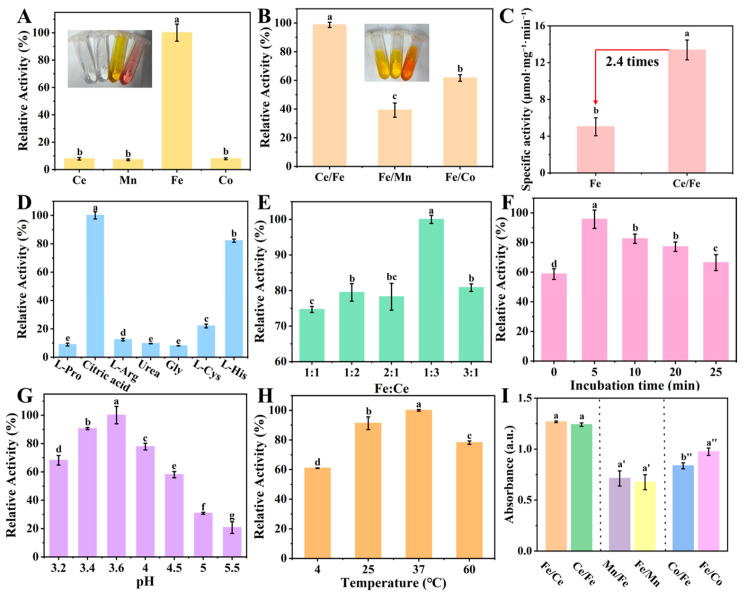
Optimization of the catalytic activity of Ce-Fe oxide. The comparison of POD activity between single metal (**A**) and bimetal (**B**), and the comparison of specific activity between Fe oxide and Ce/Fe oxide (**C**), relative activity of Ce-Fe oxide at different ligand (**D**), ratios of Fe to Ce (**E**), incubation time (**F**), pH (**G**) and temperature (**H**), the influence of addition sequence on the POD activity (**I**). Data are presented as mean ± SD (n = 3). Different letters above the bars indicate significant differences (*p* < 0.05) as determined by one-way ANOVA followed by Tukey’s HSD post hoc test.

DESs constitute a class of homogeneous liquid systems formed by the combination of hydrogen bond donors and acceptors. Their remarkable ability to significantly depress the melting points of the constituent components is primarily attributed to the disruption of crystalline lattices through extensive hydrogen-bonding interactions, resulting in a substantial reduction in lattice energy. DESs formed by various metals exhibit different color states. In this study, DESs of Ce, Mn, Fe, and Co single metals were synthesized, presenting clear and transparent states of white, white, yellow, and pink, respectively (Figure 2A). In parallel, the POD activities of the prepared materials were systematically evaluated. Among them, the Fe oxide nanozyme exhibited the most pronounced catalytic performance and was therefore identified as the prime candidate for further investigation. To elucidate the influence of heterometallic doping on the catalytic behavior of the Fe-centric system, bimetal DESs formulations—namely, Ce/Fe, Fe/Mn, and Fe/Co—were synthesized and characterized. As shown in Figure 2B, the three solutions presented transparent states of yellow, yellow, and orange-yellow, respectively. Absorbance values indicated that the absorbance of Ce/Fe-DESs was the highest, which may be attributed to the addition of Ce, possibly promoting the electron transfer between Fe^2+^ and Fe^3+^. However, direct measurements (e.g., XPS analysis) would be required to confirm this electron transfer mechanism. Additionally, we compared the POD activities of single and bimetallic metals and found that the POD activity of Ce/Fe was approximately twice that of Fe (Figure S2). And the specific activity of Ce-Fe oxide was determined to be 13.4 ± 1.1 μmol·mg^−1^·min^−1^, which was significantly higher than that of Fe oxide (5.0 ± 1.0 μmol·mg^−1^·min^−1^, *p* < 0.01, Student’s t-test), corresponding to a 2.4-fold improvement.

To maximize the POD activity of Ce-Fe oxide, the ligand types, the molar ratio of Ce to Fe, catalytic time, pH and temperature were optimized. During ligand optimization, different amino acids, citric acid and urea were selected as HBD. Previous studies have demonstrated that carboxyl groups can serve as binding sites for hydrogen peroxide (H_2_O_2_), facilitating substrate enrichment and catalytic reactions [26]. Specifically, theoretical calculations have shown that neighboring carboxyl groups aid H_2_O_2_ adsorption and free radical generation [27]. Thus, the three carboxyl groups of citric acid provide multiple H_2_O_2_ binding sites, accounting for the highest POD activity observed in Figure 2D. Viscosity, as a critical physical property of DESs, affects mass transfer and molecular diffusion. Higher viscosity, arising from an extensive hydrogen-bond network, tends to reduce pore volume and constrain molecular diffusion, which may hinder Apt adsorption. Therefore, DESs with lower viscosity are expected to facilitate more efficient mass transport and better accessibility of binding sites, promoting Apt adsorption [28]. Among the tested ligands, citric acid exhibited the lowest viscosity (Table S1), which likely enables the most efficient Apt adsorption. This provides a rational explanation for why citric acid-based DESs showed the highest POD activity (Figure 2D) and were consequently selected as the optimal ligand.

In the optimization of the molar ratio of Fe and Ce, when Fe:Ce = 1:3, the catalytic activity was the highest. Additionally, based on the relative intensity as an indicator, the catalytic time was optimized, and the final catalytic time was optimized to 5 min, indicating that the biosensor constructed with Ce-Fe oxide can be used for rapid detection of the target substance (Figure 2F). The pH and catalytic temperature were also optimized (Figure 2G,H) [29]. Finally, when the pH was 3.6 and the catalytic temperature was 37 °C, the POD activity of Ce-Fe oxide reached the highest level. Moreover, the influence of the addition sequence of the bimetal elements on DESs was verified, and it was found that the addition sequence had little effect on the POD activity of Ce-Fe oxide (Figure 2I).

### 3.3. Structural Characterization

**Figure 3:**
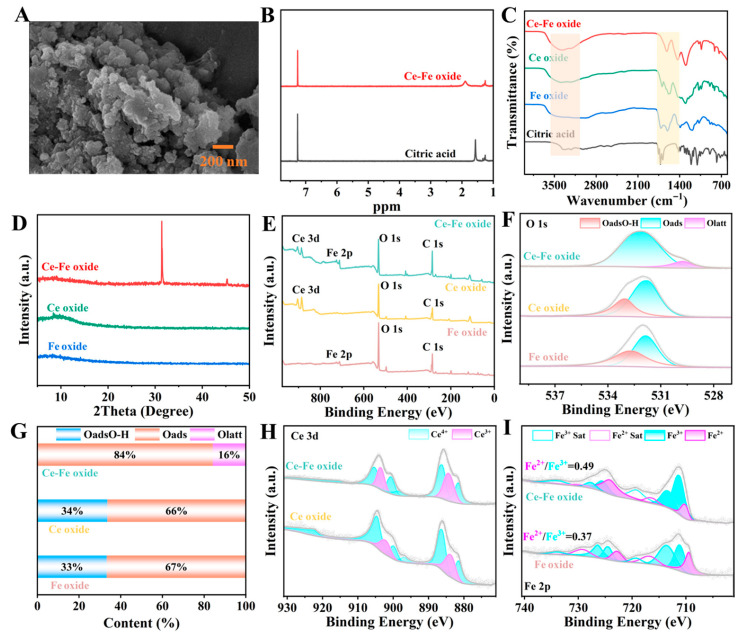
Characterization of different oxides. (**A**) SEM images, (**B**) H^1^ NMR, (**C**) FTIR spectra, (**D**) XRD patterns, (**E**) XPS spectra and (**F**) high-resolution XPS spectra of O 1s for Ce-Fe oxide, Ce oxide and Fe oxide, (**G**) O_ads_O-H, O_ads_, and O_latt_ contents distribution from XPS, (**H**) the fitted Ce 3p XPS spectra, (**I**) the fitted Fe2p XPS spectra.

The morphology of the as-synthesized Ce-Fe oxide was characterized by scanning electron microscopy (SEM), as shown in Figure 3A. The sample presents a hierarchical aggregated structure, which is assembled from numerous primary nanoparticles. Statistical analysis of the primary particles reveals that their sizes are distributed in the range of 20–80 nm, with an average diameter of approximately 45 ± 10 nm. These primary nanoparticles further aggregate into irregular secondary clusters with sizes ranging from 100 nm to 500 nm. This nanostructured architecture with a high specific surface area can provide abundant active sites, which is conducive to enhancing the POD activity of the material and improving the sensitivity of the subsequent colorimetric biosensing platform for *E. coli* O157:H7 detection.

Hydrogen nuclear magnetic resonance (^1^H NMR) spectroscopy was utilized to characterize the structural features and formation mechanism of the Ce-Fe oxide nanocomposite, as illustrated in Figure 3B. The ^1^H NMR spectrum revealed a discernible downfield shift in the intramolecular hydrogen signal, indicative of reduced electron density around the proton due to the formation of intermolecular hydrogen bonds. Specifically, upon formation of the DESs, the hydroxyl proton signal of citric acid shifted from 1.54 ppm to 1.9 ppm. This shielding effect can be attributed to the engagement of O^-^ in citric acid as a hydrogen bond acceptor, which reduces the electron density of the HBD and results in the observed downfield shift. The significant hydrogen signal shift in Ce-Fe oxide, relative to the initial reactants, provides strong evidence for the successful formation of a DES-mediated structure, confirming the critical role of hydrogen bonding in the synthesis process. Concurrently, Ce oxide and Fe oxide were prepared using the same methodology. Fourier transform infrared (FT-IR) spectroscopy was conducted to investigate the structural evolution and chemical interactions within the DES system containing Ce oxide, Fe oxide, and Ce-Fe oxide composite. Characteristic absorption peaks representing C=O groups in the 1400–1700 cm^−1^ range, similar to those of citric acid, were still observed in all three DESs (Figure 3C). In contrast, citric acid was dominated by a broad absorption band in the 3250–3400 cm^−1^ region, which was characteristic of O-H stretching vibrations and indicative of extensive hydrogen bonding. Following DES formation, the hydroxyl absorption peak shifted, and its width changed, indicating hydrogen bond formation during the process. This alteration in absorption peaks for certain hydrogen-containing functional groups confirmed the successful preparation of the DESs.

Powder X-ray diffraction (XRD) analysis of the three metal oxides revealed that the Ce-Fe oxidec oxide exhibited sharper XRD peaks and higher crystallinity compared to the monometallic oxides (Figure 3D). X-ray photoelectron spectroscopy (XPS) characterization of elemental distribution in both single- and double-metal oxides revealed that the total spectrum of Ce-Fe oxide exhibited characteristic peaks for both Ce and Fe, confirming successful synthesis (Figure 3E). This result also correlated with elemental mapping observed in the scanning electron microscope (SEM) (Figure S3). The C 1s spectra confirmed the presence of C=O, C-O, and C-C bonds in all three metal oxides (Figure S4). The O 1s spectrum of the Fe oxide exhibited two peaks at 531.87 and 532.66 eV, corresponding to surface-adsorbed oxygen (O-, O^2−^, or O_2_^2−^) (O_ads_) and surface hydroxyl oxygen (O_ads_O-H) (Figure 3F) [30]. With Ce addition, the Fe oxide altered the coordination environment of O atoms, causing the original O_ads_ to disappear and being replaced by O_ads_ at 532.13 eV and lattice oxygen O_latt_ at 529.77 eV. Compared to Ce oxide and Fe oxide alone, the peak area ratios of O_latt_/O_total_ (0.16 vs. 0.06) and O_ads_/O_total_ (0.84 vs. 0.66, 0.84 vs. 0.67) both increased (Figure 3G). XPS analysis revealed that Ce doping effectively increased the concentration of surface oxygen vacancies and adsorbed active oxygen species in the Ce-Fe oxide. These oxygen species are widely recognized as critical active sites and precursors for the generation of reactive oxygen species (ROS) during POD-mimicking catalysis. Although direct in situ ROS detection was not performed in this work, the significantly enhanced POD activity and improved colorimetric sensing performance for *E. coli* O157:H7 strongly support the hypothesis that Ce doping promotes ROS generation. Future work will focus on direct ROS quantification via electron spin resonance (ESR) spectroscopy and fluorescent probe assays to further validate this mechanism.

The high-resolution XPS spectrum of the Ce 3d energy band (Figure 3H) was fitted into eight peaks. Six of these peaks can be attributed to Ce^4+^, and the other two to Ce^3+^, indicating the coexistence of Ce^3+^ and Ce^4+^ mixed valence states in Ce oxide, with Ce^4+^ being predominant. High-resolution XPS analysis of Fe (Figure 3I) revealed characteristic peaks for Fe^2+^ and Fe^3+^ in both Ce-Fe oxide and Fe oxide, accompanied by distinct satellite peaks, confirming the mixed valence states of Fe. This coexistence is consistent with the presence of redox-active iron centers, which may facilitate electron transfer during the TMB-H_2_O_2_ reaction. It is widely accepted that the reversible Fe^2+^/Fe^3+^-mediated redox cycle is a primary mechanism underlying the POD activity of metal oxides [31]. In this cycle, Fe^2+^ reacts with H_2_O_2_ to generate ROS, which subsequently oxidize TMB. Compared with the monometallic Fe oxide, the Fe^2+^/Fe^3+^ ratio in Ce-Fe oxide increased from 0.37 to 0.49, which we hypothesize is due to Ce doping accelerating electron transfer from Fe^3+^ to Fe^2+^. Thermodynamically, Fe^2+^ can decompose H_2_O_2_ into ·OH based on the redox potentials of Fe^3+^/Fe^2+^ (0.77 V) and H_2_O_2_/H_2_O (1.776 V) [32]. However, because the redox potential of Ce^3+^/Ce^4+^ (1.72 V) is close to that of H_2_O_2_/H_2_O, the catalytic process of H_2_O_2_ generating ·OH at Ce^3+^ sites is kinetically very slow [33]. Therefore, it is proposed that Ce^3+^ primarily functions as a substrate adsorption site rather than a catalytic site.

### 3.4. The POD Activity Analysis of Ce-Fe Oxide

**Figure 4:**
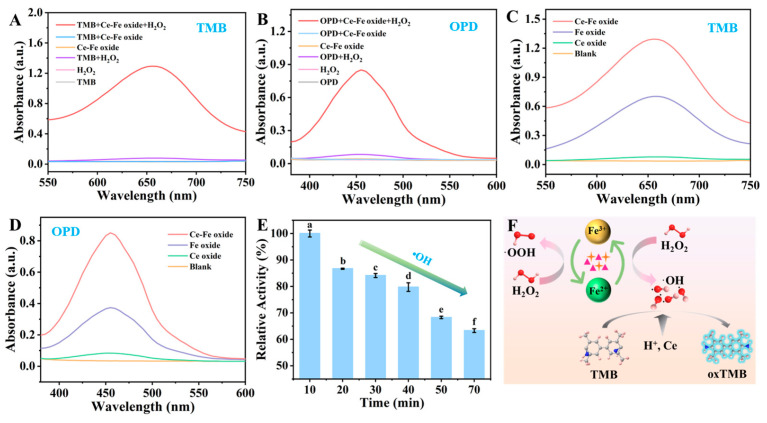
Study on the POD activity of Ce-Fe oxides. Absorption spectra of solutions containing TMB (**A**) and OPD (**B**) as catalytic substrates, comparison of POD activity of Ce-Fe oxide, Ce oxide and Fe oxide using the TMB probe (**C**) and OPD probe (**D**), (**E**) verification of the generation of ·OH for Ce-Fe oxide under MB substrate conditions, (**F**) the mechanism of TMB oxidation catalyzed. Data are presented as mean ± SD (n = 3). Different letters above the bars indicate significant differences (*p* < 0.05) as determined by one-way ANOVA followed by Tukey’s HSD post hoc test.

To verify the POD activity of the prepared Ce-Fe oxide, enzymatic substrates including TMB and o-phenylenediamine (OPD) were added. The respective substrates were introduced into the reaction mixtures under varying experimental conditions. As depicted in Figure 4A, a pronounced characteristic absorbance peak at 652 nm, corresponding to oxidized TMB (oxTMB), was exclusively observed in the presence of both H_2_O_2_ and the Ce-Fe oxide nanozyme, confirming the essential role of the nanocomposite in catalyzing the chromogenic reaction. However, the Ce-Fe oxide solution exhibited negligible absorbance in the absence of H_2_O_2_. This indicated that Ce-Fe oxide exhibited POD activity rather than oxidase activity, and also demonstrated that H_2_O_2_ was an indispensable component for Ce-Fe oxide to generate POD activity. In comparative experiments, we also measured TMB, H_2_O_2_, Ce-Fe oxide, and TMB+H_2_O_2_ individually, none of which showed significant changes in absorbance. Similar absorbance changes were observed for OPD (Figure 4B). These results all indicated that Ce-Fe oxide had POD activity.

When H_2_O_2_ was present, the implementation process of the color reaction using the POD substrates TMB and OPD was carried out to study the similar POD activity of Ce-Fe oxide, Fe oxide, and Ce oxide [34]. As shown in Figure 4C, in the presence of H_2_O_2_, Ce-Fe oxide and Fe oxide as catalysts caused a significant absorption peak at 652 nm in the UV-Vis spectrum of TMB, indicating that Ce-Fe oxide and Fe oxide can oxidize TMB in the presence of H_2_O_2_. Similarly, with OPD as the substrate, in the presence of H_2_O_2_, Ce-Fe oxide and Fe oxide as catalysts caused a significant absorption peak at 452 nm in the UV-Vis spectrum of OPD, indicating that Ce-Fe oxide and Fe oxide can oxidize OPD in the presence of H_2_O_2_ (Figure 4D). However, adding Ce oxide only resulted in weak absorption peaks at 652 nm (TMB) and 452 nm (OPD), indicating that Ce oxide did not exhibit similar POD activity. From the comparison experiments of absorbance, it can also be seen that the POD activity of Ce-Fe oxide was approximately twice that of Fe oxide. This result indicated that the POD activity of the bimetal system was indeed higher than that of a single metal.

Further, steady-state kinetic assessment was conducted using the Michaelis–Menten model analysis, from which the values of the K_m_ and the V_max_ could be obtained directly from the Lineweaver–Burk plots. The K_m_ reflects the substrate affinity; the lower the value, the higher the affinity. The value of Vmax is positively correlated with the biological catalytic activity. Similar analyses were performed on Ce-Fe oxide and Fe oxide to determine the values of K_m_ and V_max_. The K_m_ of the Fe oxide solution for TMB and H_2_O_2_ was 0.776 ± 0.2 mM and 3.441 ± 0.9 mM, respectively, and the V_max_ was (44.2 ± 8.2) × 10^−8^ M/s and (15.8 ± 0.7) × 10^−8^ M/s (Figure S5). For the Ce-Fe oxide solution, the K_m_ for TMB and H_2_O_2_ was only 0.333 ± 0.03 mM and 0.184 ± 0.006 mM, respectively, and the V_max_ was (78.8 ± 3.7) × 10^−8^ M/s and (4.83 ± 0.03) × 10^−8^ M/s (Figure S6). As shown in Table S2, the POD activity of Ce-Fe oxide was significantly stronger than that of Fe oxide. Compared with natural enzymes, it also exhibited enhanced affinity for H_2_O_2_ and TMB [35]. Meanwhile, compared with other metal oxide nanozymes, when H_2_O_2_ and TMB were used as variables, Ce-Fe oxide exhibited comparable affinity [36,37,38,39,40,41,42]. Therefore, the introduction of Ce oxide led to an enhancement in the POD activity of Ce-Fe oxide.

In order to further explore and verify the reaction intermediates generated during the catalytic oxidation of TMB by Ce-Fe oxide, the probe of benzoic acid (BA) was used. In the presence of ·OH, the probe can be converted into a fluorescent product, hydroxybenzoic acid. As shown in Figure S7, the fluorescence intensity measured at 405 nm verified that Ce-Fe oxide has similar POD activity. At the same time, the degradation of different concentrations of methylene blue (MB) at 664 nm confirmed the stability of Ce-Fe oxide in the presence of oxygen (Figure 4E). Based on this and previous XPS analysis, a mechanism for the oxidation of TMB by Ce-Fe oxide in the presence of H_2_O_2_ was proposed (Figure 4F). Firstly, H_2_O_2_ is adsorbed on the surface of Ce-Fe oxide. Subsequently, Fe^2+^ in Ce-Fe oxide converted H_2_O_2_ into ·OH through a process similar to the Fenton reaction (Equation (3)) [43]. Then, Fe^3+^ converted H_2_O_2_ into·OOH (Equation (4)). Finally, under acidic conditions, the colorless TMB molecules were oxidized by ·OH, thereby forming the oxTMB (Equation (5)) [44]. For Fe oxide, although the above reaction can also be achieved, the amount of H_2_O_2_ was much lower than that of Ce-Fe oxide. A plausible explanation is that the addition of Ce increases the adsorption capacity of Ce-Fe oxide for H_2_O_2_, thereby facilitating electron transfer between Fe^2+^ and Fe^3+^. This could generate more ·OH and consequently more oxTMB.

(3) Fe^2+^ + H_2_O_2_ → Fe^3+^ +·OH + OH^−^

(4) Fe^3+^ + H_2_O_2_ → Fe^2+^ +·OOH + H^+^

(5) ·OH + H^+^ + TMB → H_2_O + oxTMB

### 3.5. Analytical Performance of the Proposed Colorimetric Biosensor

**Figure 5:**
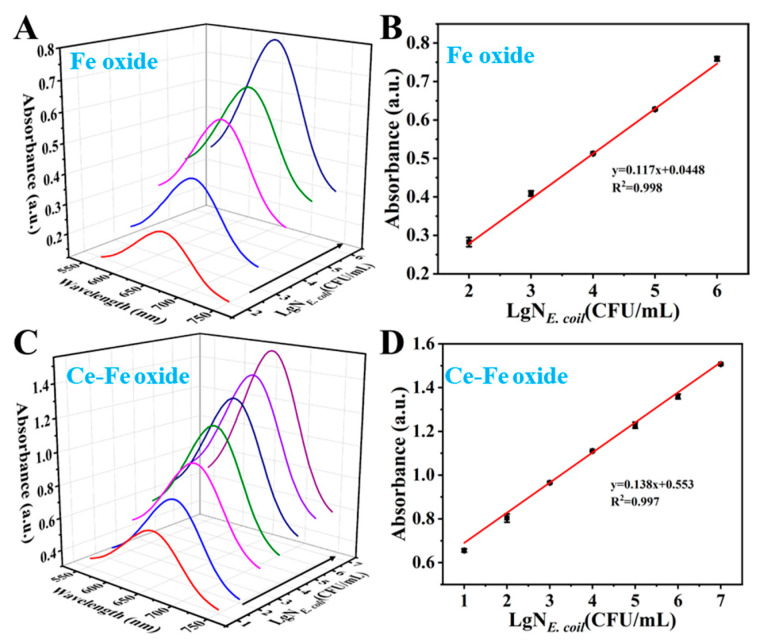
Comparison of calibration curves. (**A**) UV spectra of the sensing system based on Fe oxide and (**B**) linear analysis of absorbance at 652 nm with different concentrations of *E. coli* O157:H7, (**C**) UV spectra of the sensing system based on Ce-Fe oxide and (**D**) linear analysis of absorbance at 652 nm with different concentrations of *E. coli* O157:H7.

To improve the detection performance of the proposed colorimetric biosensor, the concentration of MBs and Ce-Fe oxide was optimized (Figure S8). Then, under the optimal conditions where the concentration of MBs and the concentration of Ce-Fe oxide were both 0.8 mg/mL, the performance of the sensors based on Fe oxide and Ce-Fe oxide was evaluated. As illustrated in Figure 5A, the Fe oxide-based biosensor exhibited a concentration-dependent increase in absorbance, attributable to the enhanced formation of capture complexes between Fe oxide@Apt, MBs@Apt and the target bacteria. The corresponding calibration curve (Figure 5B) demonstrated a strong linear relationship between the absorbance at 652 nm and bacterial concentrations ranging from 10^2^ to 10^6^ CFU/mL, following the regression equation y = 0.117x + 0.0448 (R^2^ = 0.998). The LOD was determined to be 10^2^ CFU/mL. Similarly, the Ce-Fe oxide-based biosensor showed a pronounced increase in absorbance at 652 nm with escalating bacterial concentrations (Figure 5C). This system exhibited a wide linear detection range from 10^1^ to 10^7^ CFU/mL, described by the equation y = 0.138x + 0.553 (R^2^ = 0.994), and achieved a significantly lower LOD of 10^1^ CFU/mL, underscoring its enhanced sensitivity over the Fe oxide-based biosensor (Figure 5D). After comparison, the biosensor based on Ce-Fe oxide had a wider linear range and lower detection limit than the biosensor based on Fe oxide, and had higher sensitivity. Compared with other studies [25,45,46,47,48,49,50,51], the biosensor based on Ce-Fe oxide had comparable sensitivity (Table S3).

**Figure 6:**
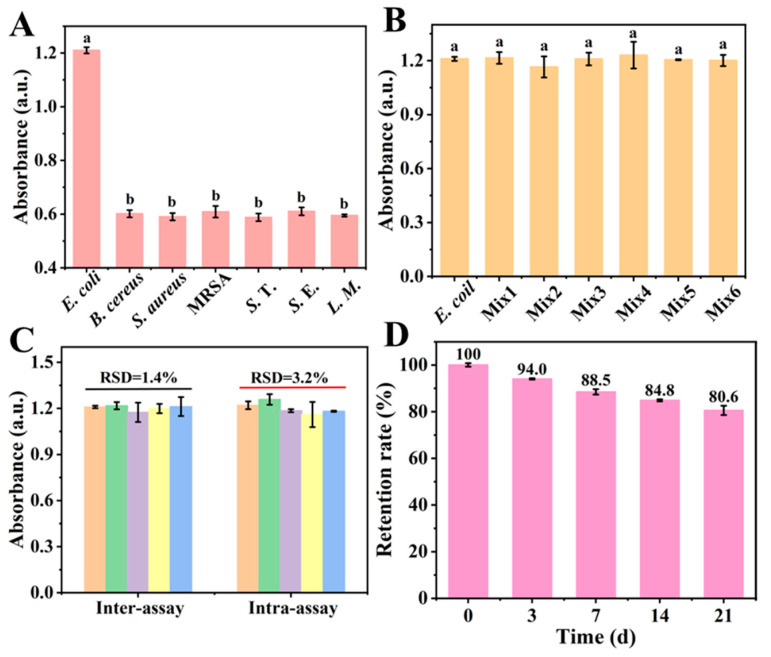
Evaluation of sensor sensitivity. (**A**,**B**) Specificity study, (**C**) repeatability test and (**D**) stability test. Data are presented as mean ± SD (n = 3). Different letters above the bars indicate significant differences (*p* < 0.05) as determined by one-way ANOVA followed by Tukey’s HSD post hoc test.

To evaluate the specificity of the biosensor, *Bacillus cereus* (*B. cereus*), *Staphylococcus aureus* (*S. aureus*), Methicillin-resistant *Staphylococcus aureus* (MRSA), *Salmonella typhimurium* (*S.* T.), *Salmonella* enteritidis (*S.* E.) and *Listeria monocytogenes* (*L. M.*) were selected as interfering strains. And the detailed sources of the bacterial strains were shown in Table S4. Even when the concentration of the interfering strains was 100 times that of the target strains, the absorbance was still much lower than that of *E. coli* O157:H7 (Figure 6A). At the same time, when *E. coli* O157:H7 and the interfering strains were mixed, the absorbance under the mixed bacterial solution was not significantly different from that of the *E. coli* O157:H7 alone (Figure 6B). In conclusion, the biosensor based on Ce-Fe oxide had acceptable selectivity. However, it should be noted that the panel of interfering strains, while covering several common foodborne pathogens, does not fully represent the complex microbial communities present in real food matrices such as diverse background bacteria, yeasts, and molds. Therefore, the selectivity demonstrated here is a preliminary proof-of-concept; further validation using a broader range of food-relevant microorganisms would be necessary to assess the sensor’s performance in authentic food environments.

For biosensors aimed at the detection of target bacterial pathogens, the repeatability of the proposed method is crucial. The relative standard deviations (RSD) for inter-assay and intra-assay repeatability were 1.4% and 3.2%, respectively. These values were obtained from five independent batches of sensors (biological replicates), with three replicate measurements per batch (technical replicates) using a Ce-Fe oxide concentration of 0.8 mg/mL and an *E. coli* O157:H7 concentration of 10^5^ CFU/mL. All measurements were performed on independently prepared sensors. These results indicate that the developed biosensor exhibits acceptable repeatability (Figure 6C).

To further assess the operational stability, Ce-Fe oxide was stored at 4 °C, and its absorbance response was monitored at weekly intervals over a period of 3 weeks. After 3 weeks of storage, the absorbance response remained above 80%, demonstrating good stability (Figure 6D). These results highlighted the long-term usage potential of this biosensor in field detection applications. This observed stability was primarily attributed to the exceptional inherent stability of the synthesized Ce-Fe oxide-based DES biosensor. The demonstrated long-term stability of the biosensor ensured reliable and continuous monitoring of foodborne pathogens, rendering it highly suitable for sustained quality assurance throughout food processing and storage cycles. Furthermore, the system exhibited exceptional sensitivity and rapid response, enabling the early detection of microbial contamination, which significantly reinforced food safety management and consumer protection. The integration of these robust performance characteristics offers a cost-effective, efficient, and readily scalable platform for real-time pathogen surveillance in food safety applications.

### 3.6. Detection of E. coli O157:H7 in Spiked Samples

To further assess the analytical performance of the developed colorimetric biosensors in real samples, the Fe oxide-based and Ce-Fe oxide-based systems were employed for the detection of *E. coli* O157:H7, including lake water, orange juice, and lettuce extracts, using the standard addition method. Specifically, *E. coli* O157:H7 standard solutions were introduced into the samples to prepare artificially contaminated samples at final concentrations of 10^2^, 10^3^ and 10^4^ CFU/mL. As shown in Table 1, the RSD range of the biosensor based on Fe oxide was 1.6–8.8%, and the recovery rate range of the three samples was 99–103%. The RSD range of the biosensor based on Ce-Fe oxide was 1.2–6.7%, and the recovery rate range of the three samples was 89–104%. The recovery as low as 89% is likely due to matrix effects from the complex sample matrices, which may partially suppress the catalytic activity or recognition efficiency. Nevertheless, the recovery range is acceptable for preliminary validation. Although the recovery rate of the biosensor based on Fe oxide was closer to 100% and had higher accuracy, the RSDs of the biosensor based on Ce-Fe oxide were lower. In conclusion, these acceptable RSDs and recovery rate values indicate the feasibility of the DES solutions in the detection of spiked samples in this study.

## 4. Conclusions

Here, a bimetal oxide nanozyme, Ce-Fe oxide, was successfully prepared using DESs. The synthesis was carried out at 80 °C under ambient pressure. By adjusting the metal types and ligands, a Ce-Fe oxide solution with strong hydrogen-bond interactions was obtained. Modulating the metal valence state and oxygen vacancy content as two key factors significantly enhanced the POD activity of the nanozyme. Compared with the single-metal oxide Fe oxide, the addition of Ce may promote electron transfer between Fe^2+^ and Fe^3+^, potentially generating more ·OH radicals and thus leading to increased oxidation of TMB. Both Fe oxide and Ce-Fe oxide were successfully integrated into a colorimetric biosensor for the detection of *E. coli* O157:H7. The LOD of the Ce-Fe oxide-based biosensor was determined to be 10^1^ CFU/mL, which is lower than that of the Fe oxide-based biosensor (10^2^ CFU/mL), indicating improved sensitivity. In spiked food matrix samples, both sensors exhibited satisfactory recovery rates and RSD, demonstrating the feasibility of this approach as a preliminary proof-of-concept detection platform for *E. coli* O157:H7 under sterilized and spiked matrix conditions. Further validation using naturally contaminated samples and without a pre-enrichment step would be required to support practical field applications.

## Figures and Tables

**Table 1:** Application of a colorimetric biosensor based on Fe oxide and Ce-Fe oxide in spiked samples.

Spiked (CFU/mL)		Detection (CFU/mL)		Recovery (%)		RSD (%)	
		Ce-Fe Oxide	Fe Oxide	Ce-Fe Oxide	Fe Oxide	Ce-Fe Oxide	Fe Oxide
Lake water	1 × 10^2^	1.02 × 10^2^	0.91 × 10^2^	102	91	1.6	6.7
	1 × 10^3^	1.03 × 10^3^	1.00 × 10^3^	103	100	1.6	2.8
	1 × 10^4^	1.02 × 10^4^	1.01 × 10^4^	102	101	2.2	2.3
Orange juice	1 × 10^2^	1.03 × 10^2^	0.89 × 10^2^	103	89	8.8	1.4
	1 × 10^3^	0.99 × 10^3^	0.98 × 10^3^	99	98	4.1	2.4
	1 × 10^4^	1.02 × 10^4^	1.00 × 10^4^	102	100	3.1	4.2
Lettuce	1 × 10^2^	1.03 × 10^2^	0.92 × 10^2^	103	92	2.4	1.3
	1 × 10^3^	0.99 × 10^3^	0.99 × 10^3^	99	99	3.9	1.2
	1 × 10^4^	1.01 × 10^4^	1.04 × 10^4^	101	104	3.7	2.7

## Data Availability

The original contributions presented in the study are included in the article/Supplementary Materials, further inquiries can be directed to the corresponding author.

## Supplementary Materials

The following supporting information can be downloaded at: https://www.mdpi.com/article/10.3390/foods15081391/s1, Figure S1: Zeta potential of Ce-Fe oxide and Ce-Fe oxide@Apt; Figure S2: The comparison of POD activity between Fe oxide and Ce-Fe oxide; Figure S3: SEM mapping of Ce-Fe oxide; Figure S4: High-resolution XPS spectra of C 1s for Ce-Fe oxide, Ce oxide and Fe oxide; Figure S5: Kinetic analysis of Fe oxides. The kinetic curves of Fe oxide with TMB (A) and H_2_O_2_ (C) as substrates, and the corresponding double reciprocal plots of Fe oxide with TMB (B) and H_2_O_2_ (D) as substrates; Figure S6: Kinetic analysis of Ce-Fe oxides. The kinetic curves of Ce-Fe oxide with TMB (A) and H_2_O_2_ (C) as substrates, and the corresponding double reciprocal plots of Ce-Fe oxide with TMB (B) and H_2_O_2_ (D) as substrates; Figure S7: Verification of the generation of hydroxyl radicals for Ce-Fe oxide under BA substrate; Figure S8: Optimization of the experimental conditions. (A) MB concentration and (B) Ce-Fe oxide concentration; Table S1: Physicochemical properties of deep eutectic solvent after screening (n = 3); Table S2: Comparison of the enzymatic kinetic parameters; Table S3: Comparison with previous verified biosensors for *E. coli* O157:H7 detection; Table S4: The bacteria strains used for the specificity test.
