# Supplementary material for: Bimetallic Deep Eutectic Solvent-Driven Ce-Fe Oxide Nanozyme Based on Electron Transfer for the Colorimetric Detection of E. coli O157:H7 in Food
Source: Foods. 2026 Apr 16;15(8):1391. doi: 10.3390/foods15081391 (PMC13114577; doi:10.3390/foods15081391)
Supplement: Supplementary file 1 [file foods-15-01391-s001.zip › foods-4222623-supplementary.pdf]

**Supporting Information**

# Bimetallic Deep Eutectic Solvent-Driven Ce-Fe Oxide Nanozyme Based on Electron Transfer for the Colorimetric Detection of *E. coli* O157:H7 in Food

Luyang Zhao, Yang Song, Guoyang Xie and Hengyi Xu *

State Key Laboratory of Food Science and Resources, Nanchang University, Nanchang 330047, China; 357900230020@email.ncu.edu.com (L.Z.); wangsyang202011@163.com (Y.S.); 212301006@csu.edu.cn (G.X.)

* Correspondence: hengyixu@vip.163.com or hengyixu@ncu.edu.cn

**Dr. Hengyi Xu**

Tel.: +0086-791-8830-4447 (ext-9520);

Fax: +0086-791-8830-4400.

**Materials and reagents**

The aptamer sequences used for *E. coli* O157:H7 recognition was 5'-$NH_2$-CCG GAC GCT TAT GCC TTG CCA TCT ACA GAG CAG GTG TGA CGG-3', synthesized by Shanghai Sangon Biotech Co., Ltd (Shanghai, China). Carboxylated magnetic nanoparticles (180 nm) was purchased from Allrun Nano (Shanghai, China). Luria-Bertani medium was bought from Beijing Land Bridge (Beijing, China). Agar powder, benzoic acid (BA) and methylene blue (MB) were purchased from Solarbio (Beijing, China). 3,3',5,5'-Tetramethylbenzidine (TMB) was purchased from Sangon Biotech (Shanghai, China). 30% hydrogen peroxide ($H_2O_2$), 1-Ethyl-3-(3-dimethyllaminopropyl) carbodiimide hydrochloride (EDC), nhydroxysulfosuccinimide sodium salt (NHSS), Cerium (III) nitrate hexahydrate $Ce(NO_3)_3$•$6H_2O$ and Dimethyl sulfoxide (DMSO) were purchased from Aladdin (Shanghai, China). Iron chloride hexahydrate and 20×phosphate buffered saline (20×PBS) buffer solution were all provided by Macklin (Shanghai, China). Sodium hydroxide (NaOH) and citric acid was from Sinopharm (Shanghai, China). Anhydrous sodium acetate and acetic acid were purchased from Xilong Scientific (Shantou, China). Ultrapure water used in all experiments was from the Milli-Q system (Millipore, USA). All glassware was soaked in aqua regia ($HNO_3$: HCl=1:3, v/v) for more than 2 h and then rinsed thoroughly with ultrapure water. All chemicals were at least analytical reagent grade.

**Apparatus**

The morphology of Ce-Fe oxide was investigated using a Scanning Electron Microscope (SEM) (Regulus 8100, Hitachi, Japan). Powder X-ray Diffraction (XRD) measurements were performed using a diffractometer (Bruker D8 Advance, Germany). Surface chemical elements were tested by X-ray photoelectron spectroscopy (Thermo Scientific K-Alpha, USA). The chemical functional groups were characterized by the Fourier transform infrared spectroscopy (FT-IR) (Thermo Fisher Scientific Nicolet iS20, USA). The UV-vis absorbance spectra were obtained by a microplate S-4 reader (Varioskan multimode microplate reader, Thermo Fisher Scientific, Waltham, USA).

**Culture of experimental strains**

*Escherichia coli* O157:H7 (*E. coli* O157:H7), *Staphylococcus aureus (S. aureus), Salmonella* typhimurium *(S.* T.*), Listeria monocytogenes (L. M.), Salmonella* enteritidis *(S.* E.*),* Methicillin-resistance Staphylococcus aureus (MRSA) and *Bacillus cereus (B. cereus)* were respectively cultured in sterile Lysogeny-Broth (LB) medium (containing 10 g/L tryptone, 5 g/L yeast extract, 10 g/L sodium chloride) in a shaking incubator at 37°C until they reached the stable growth stage. After overnight culture, 1 mL of each bacterial cell suspension was taken and centrifuged at 7000 rpm for 20 min. Then, it was washed 3 times with PBS (0.01 mol/L, pH=7.4) and re-suspended with 1 mL PBS. The concentration of bacterial cell suspensions could be obtained by measuring the concentration of bacterial cell suspensions on LB plates using the plate counting method. Different concentrations of bacterial cell suspensions could be obtained by dilution with PBS buffer.

**Optimization of catalytic conditions**

The effects of incubation time, pH and temperature on the POD activity of Ce-Fe oxide were investigated by using TMB as a typical chromogenic substrate [29]. To optimize the reaction time, the mixture of Ce-Fe oxide TMB, $H_2O_2$ and NaAc buffer (pH 3.6) was incubated at 37°C for different times (0, 5, 10, 15, 25 min). To explore the influence of pH on the catalytic activity of the nanozyme, 190 μL of NaAc buffer with different pH values (pH 3.2, 3.4, 3.6, 4.0, 4.5, 5.0, 5.5) were taken, and 25 μL of 0.25 mM TMB, 10 μL of 80 μg/mL Ce-Fe oxide, and 25 μL of 5 mM $H_2O_2$ solution were added. Then, the mixture was incubated at 37°C for 5 min. After that, the absorbance of the different incubation samples at 652 nm was measured. Subsequently, under the optimal pH conditions, the effect of incubation temperature (4, 25, 37, 60°C) on the catalytic activity of Ce-Fe oxide was investigated. Similarly, the mixture containing Ce-Fe oxide, TMB, $H_2O_2$ and NaAc buffer (pH=3.6) was reacted at different temperatures, and the UV absorbance of the reaction solution at 652 nm was measured.

**Verify the POD activity of Ce-Fe oxide using TMB and OPD as substrates**

By using TMB and OPD as typical chromogenic substrates, the absorbance under

different conditions was compared. When using TMB as the substrate, the absorbance at 652 nm in different states of TMB+$H_2O_2$+Ce-Fe oxide, TMB+ Ce-Fe oxide, Ce-Fe oxide, TMB+$H_2O_2$, $H_2O_2$ and TMB was compared. At the same time, the absorbance at 625 nm for $CeO_2$/$Fe_2O_3$, $CeO_2$, and Fe oxide was also compared. Similarly, when using OPD as the substrate, the absorbance at 452 nm in OPD+$H_2O_2$+Ce-Fe oxide, OPD+Ce-Fe oxide, Ce-Fe oxide, OPD+$H_2O_2$, $H_2O_2$ and OPD was compared. At the same time, after catalysis for 5 min, the absorbance at 452 nm for Ce-Fe oxide, Ce oxide, and Fe oxide was also compared.

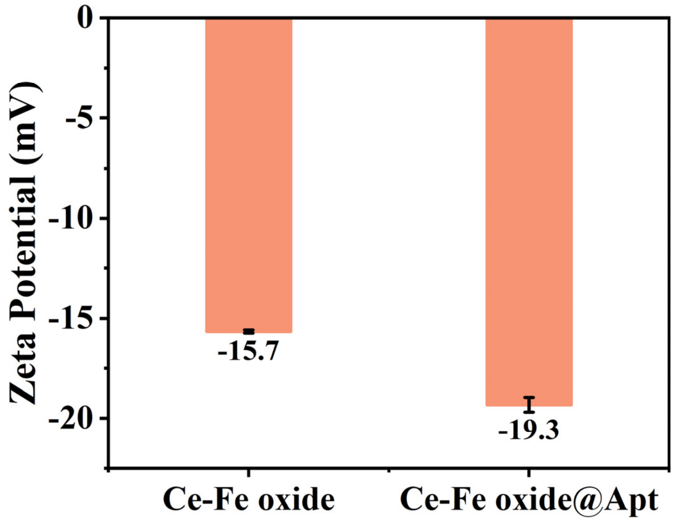


**Figure S1.** Zeta potential of Ce-Fe oxide and Ce-Fe oxide@Apt.

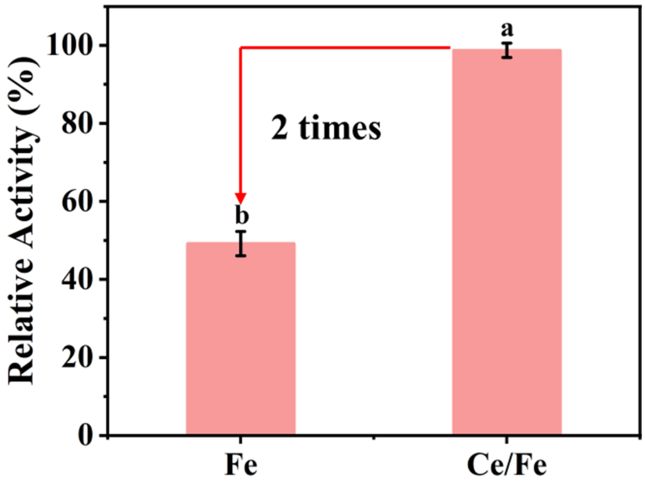


**Figure S2.** The comparison of POD activity between Fe oxide and Ce-Fe oxide.

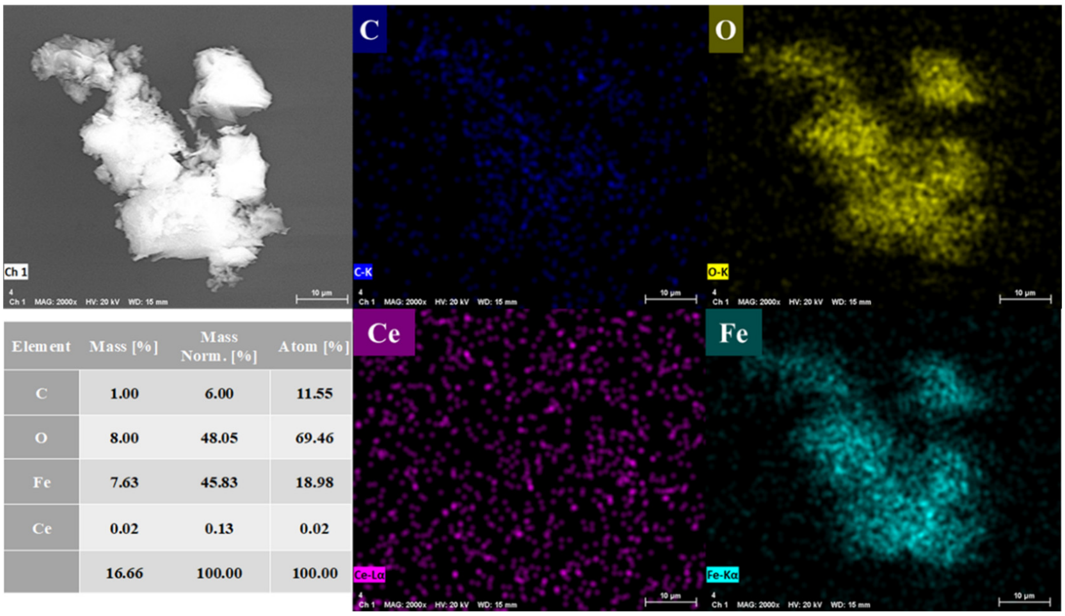


**Figure S3.** SEM mapping of Ce-Fe oxide.

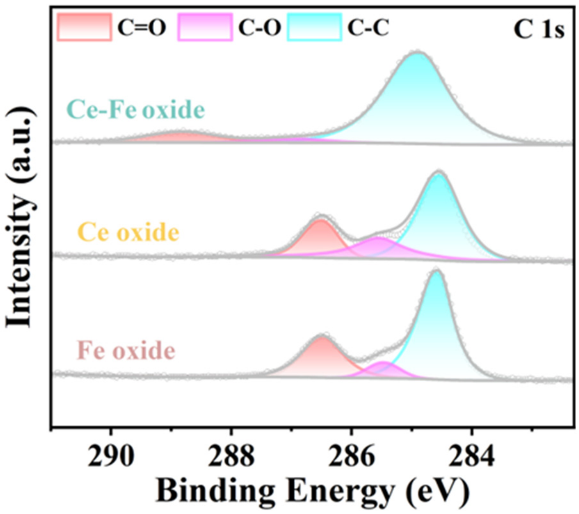


**Figure S4.** High-resolution XPS spectra of C 1s for Ce-Fe oxide, Ce oxide and Fe oxide.

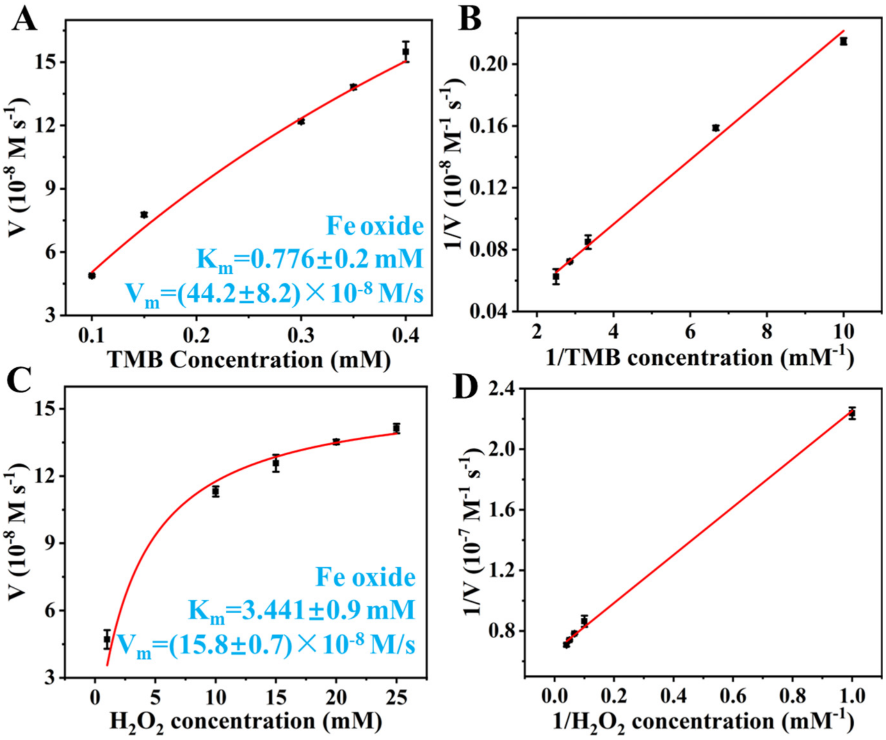


**Figure S5.** Kinetic analysis of Fe oxides. The kinetic curve (A) of Fe oxide with TMB as the variable and the corresponding double reciprocal plots (B), as well as the kinetic curve (C) with $H_2O_2$ as the variable and the corresponding double reciprocal plots (D).

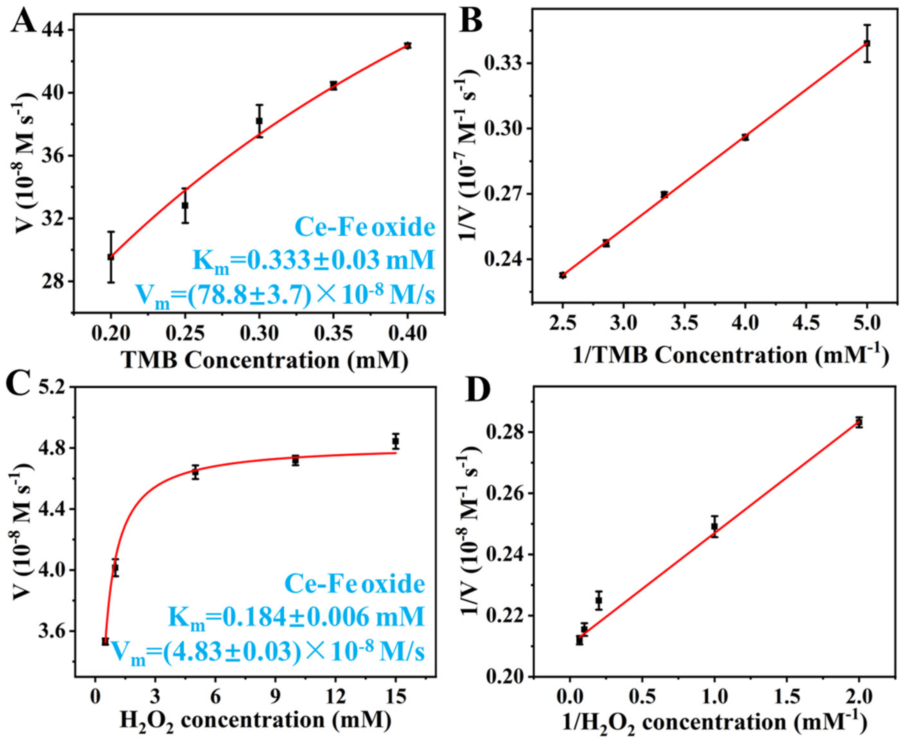


**Figure S6.** Kinetic analysis of Ce-Fe oxides. The kinetic curves of Ce-Fe oxide with TMB as the variable and the corresponding double reciprocal plots (B), as well as the kinetic curve (C) with $H_2O_2$ as the variable and the corresponding double reciprocal plots (D).

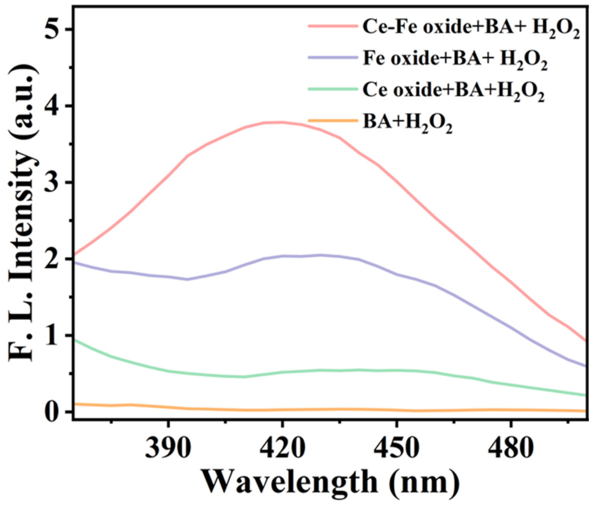


**Figure S7.** Verification of the generation of hydroxyl radicals for Ce-Fe oxide under BA substrate.

**Optimization of detection experiment conditions**

To improve the detection performance of the proposed colorimetric biosensor, the

key reaction conditions was optimized, including the concentration of MBs and Ce-Fe oxide, based on the absorbance at 652 nm as the indicator. As shown in Fig. S7A, the absorbance gradually increased with the increase of the concentration of MBs. This was because as the concentration of MBs increased, the Apt connected to it also increased, and the sandwich structure formed with *E. coli* O157:H7 also increased. Subsequently, the absorbance reached its maximum at 0.8 mg/mL of MBs, this was because the Apt molecules loaded on the MBs had reached saturation. As for the optimization of Ce-Fe oxide, within the concentration range of 0.2-0.8 mg/mL, the absorbance increased as the concentration of Ce-Fe oxide increased (Fig. S7B). Until the concentration of Ce-Fe oxide reached 0.8 mg/mL, the Apt on Ce-Fe oxide reached saturation. After that, as the concentration of Ce-Fe oxide increased, the absorbance remained unchanged. Therefore, the optimal experimental conditions were set as 0.8 mg/mL of MBs and 0.8 mg/mL of Ce-Fe oxide concentration, and the subsequent experiments were conducted.

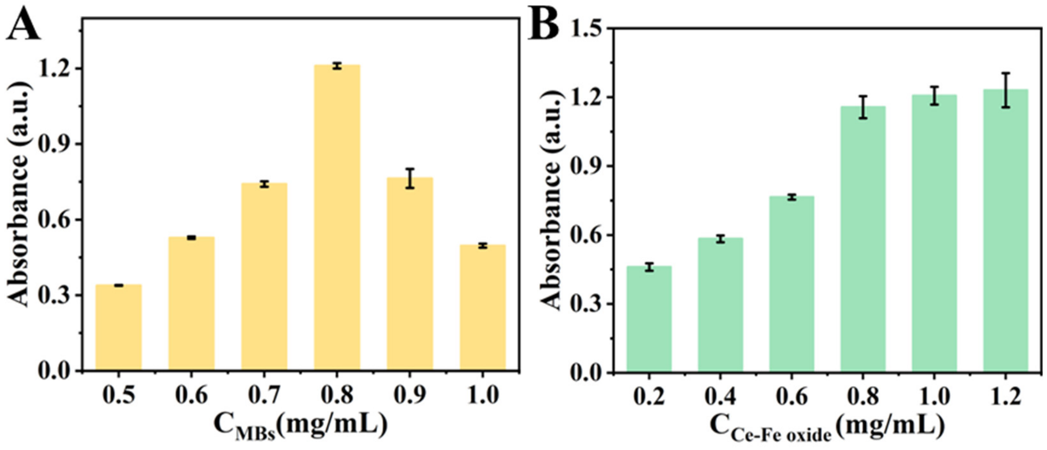


**Figure S8.** Optimization of the experimental conditions. (A) MBs concentration and (B) Ce-Fe oxide concentration.

**Table S1.** Physicochemical properties of deep eutectic solvent after screening (n=3).

| Ligand | L-Pro | Citric acid | L-Arg | Urea | Gly | L-Cys | L-His |
|---|---|---|---|---|---|---|---|
| Viscosity (Pa·s) | 2 | 0.108 | 0.151 | 0.643 | 0.163 | 0.834 | 2.3 |

**Table S2.** Comparison of the enzymatic kinetic parameters.

| Materials | $K_m$ (mM) | | $V_{max}$ ($10^{-8}$M/s) | | References |
|---|---|---|---|---|---|
| | TMB | $H_2O_2$ | TMB | $H_2O_2$ | |
| Natural enzyme (Horseradish peroxidase HPR) | 0.434 | 3.7 | 10 | 8.71 | [35] |
| $Cu_2O$ | - | 44.4 | - | 483 | [36] |
| $Co_3O_4$ nanocrystals | 0.49 | 1.9 | 16 | 12.7 | [37] |
| $Fe_2O_3$ nanoparticles | 0.439 | 120 | 4.23 | 0.17 | [38] |
| rGO@PDA@$CeO_2$ | 6.81 | 6.39 | 48.96 | 12.3 | [39] |
| Fe-$MoS_2$ | 2.82 | 0.629 | 72.89 | 8.06 | [40] |
| $Co_3O_4$-$MnO_2$ | 1.159 | - | 2.27 | - | [41] |
| Pd/$CeO_2$ | 0.227 | 4.49 | 38.2 | 32 | [42] |
| Fe oxide | 0.776 | 3.411 | 44.2 | 15.8 | This work |
| Ce-Fe oxide | 0.333 | 0.184 | 78.8 | 4.83 | |

**Table S3**. Comparison with previous verified biosensors for *E. coli* O157:H7 detection.

| Materials | Method | Linear range (CFU/mL) | LOD (CFU/mL) | Ref. |
|---|---|---|---|---|
| GNP/MWCNT | RGB value | $10^4$-$10^7$ | $8.43\times10^3$ | [45] |
| MBs | chemiluminescence immunoassay | $4.3\times10^3$-$4.3\times10^5$ | $1.2\times10^3$ | [46] |
| MBs | electrochemistry | $10^2$-$10^6$ | $2.05\times10^3$ | [47] |
| Au/Pt/$SiO_2$ NPs | electrochemistry | $3.5\times10^2$-$3.5\times10^8$ | $1.83\times10^2$ | [48] |
| R-CDs/BONs | fluorescence | $2.5\times10^1$-$2.5\times10^6$ | 25 | [49] |
| UCNPs | fluorescence | $10^3$-$10^8$ | $1.3\times10^2$ | [50] |
| GNPs | colorimetry | - | $1\times10^2$ | [51] |
| SiNPs | colorimetry | $1\times10^2$-$5\times10^5$ | 100 | [25] |
| Fe oxide | colorimetry | $10^2$-$10^6$ | $10^2$ | This work |
| Ce-Fe oxide | | $10^1$-$10^7$ | $10^1$ | |

Quartz Crystal Microbalance (QCM); Breakable Organosilica Nanocapsules (BONs); Up-conversion nanoparticles (UCNPs); Gold nanoparticles (GNPs).

**Table S4**. The bacteria strains used for the specificity test.

| No. | Bacteria strains | Source* |
|---|---|---|
| 1 | *E. coli* O157:H7 | ATCC 43888 |
| 2 | MRSA | NCTC 12493 |
| 3 | *B. cereus* | JX-CDC JDZ0102Y |
| 4 | *L. M* | ATCC 13932 |
| 5 | *S. E.* | ATCC 13076 |
| 6 | *S. T.* | CMCC 13311 |
| 7 | *S. aureus* | CMCC 26001 |

*The strains were isolated from Jiang Xi Province Center for Disease Control and Prevention.
